# Dysfunctional HCN ion channels in neurological diseases

**DOI:** 10.3389/fncel.2015.00071

**Published:** 2015-03-10

**Authors:** Jacopo C. DiFrancesco, Dario DiFrancesco

**Affiliations:** ^1^Department of Neurophysiology, Foundation Neurological Institute C. BestaMilano, Italy; ^2^Department of Neurology, San Gerardo Hospital and Laboratory of Neurobiology, Milan Center for Neuroscience, University of Milano–BicoccaMonza, Italy; ^3^The PaceLab, Department of Biosciences, University of MilanoMilano, Italy

**Keywords:** epilepsy, HCN channels, HCN mutation, I_h_ current, seizure, Parkinson’s disease, pain

## Abstract

Hyperpolarization-activated cyclic nucleotide-gated (HCN) channels are expressed as four different isoforms (HCN1-4) in the heart and in the central and peripheral nervous systems. HCN channels are activated by membrane hyperpolarization at voltages close to resting membrane potentials and carry the hyperpolarization-activated current, dubbed I_f_ (funny current) in heart and I_h_ in neurons. HCN channels contribute in several ways to neuronal activity and are responsible for many important cellular functions, including cellular excitability, generation, and modulation of rhythmic activity, dendritic integration, transmission of synaptic potentials, and plasticity phenomena. Because of their role, defective HCN channels are natural candidates in the search for potential causes of neurological disorders in humans. Several data, including growing evidence that some forms of epilepsy are associated with HCN mutations, support the notion of an involvement of dysfunctional HCN channels in different experimental models of the disease. Additionally, some anti-epileptic drugs are known to modify the activity of the I_h_ current. HCN channels are widely expressed in the peripheral nervous system and recent evidence has highlighted the importance of the HCN2 isoform in the transmission of pain. HCN channels are also present in the midbrain system, where they finely regulate the activity of dopaminergic neurons, and a potential role of these channels in the pathogenesis of Parkinson’s disease has recently emerged. The function of HCN channels is regulated by specific accessory proteins, which control the correct expression and modulation of the neuronal I_h_ current. Alteration of these proteins can severely interfere with the physiological channel function, potentially predisposing to pathological conditions. In this review we address the present knowledge of the association between HCN dysfunctions and neurological diseases, including clinical, genetic, and physiopathological aspects.

## Introduction

The class of hyperpolarization-activated cyclic nucleotide-gated (HCN) channels comprises four human isoforms (HCN1-4) and belongs to the superfamily of voltage-dependent potassium (Kv) and cyclic nucleotide-gated (CNG) channels. HCN channels are unique in that they are dually activated by voltage hyperpolarization and intracellular cAMP (DiFrancesco, 1999, 2006; Barbuti et al., 2007a; Baruscotti et al., 2010).

Since they are constitutively open at voltages near to the resting membrane potential and, being permeable to both Na^+^ and K^+^ ions, carry at these voltages an inward current, HCN channels are well equipped to control voltage-dependent mechanisms, such as neuronal excitability, and related functions.

In neurons, HCN channels carry the hyperpolarization -activated (I_h_) current. The four isoforms are differently distributed: HCN1, 2 and 4 are widely expressed and contribute to the generation of neuronal activity, while the role of HCN3 is less clear. In neurons, HCN are responsible for several important cellular functions, including the contribution to cellular excitability, and plasticity phenomena in the brain. One of the most specific functions of HCN channels is their involvement in the generation and modulation of rhythmic activity, which is typified by the role they play in the spontaneous activity and frequency control of pacemaker cells of the heart (DiFrancesco, 1993; Pape, 1996; Robinson and Siegelbaum, 2003; Barbuti et al., 2007a).

As expected from the established role of HCN channels in neurons, in the last few years new evidence has emerged linking HCN channels’ dysfunctions to different neurological disorders, in accordance with the view that altered pacemaker activity can act as a strong pathogenic mechanism.

The vast majority of such data have been obtained in the field of epilepsy (Dyhrfjeld-Johnsen et al., 2009; Baruscotti et al., 2010; DiFrancesco et al., 2011; Reid et al., 2012; Shah et al., 2013). Together with data from animal models showing that HCN dysfunction can lead to epileptogenesis, evidence is accumulating to indicate that some inheritable forms of epilepsy can be associated in human patients with *HCN* genetic mutations leading to altered I_h_ current function and increased neuronal excitability. Although a comprehensive picture of the genetic basis of epilepsy is far from complete, these new data contribute importantly to a deeper understanding of the processes responsible for causative changes in neuronal excitability.

As well as in the central nervous system (CNS), HCN channels are also widely expressed in the peripheral nervous system (PNS), where their function is important in the perception, modulation, and transmission of sensory signals, including pain. Recent data demonstrate a central role of the HCN2 isoform in the transmission of neuropathic and inflammatory pain (Emery et al., 2011).

Moreover, HCN channels are expressed in the midbrain system, where they control the oscillatory activity of dopaminergic neurons. In line with this evidence, a new potential role of HCN channels dysfunction is also emerging in the pathogenesis of Parkinson’s disease (PD).

Full knowledge of the pathogenic mechanisms underlying these debilitating neurological diseases and the role played by aberrant HCN channel function is not at hand. However, a more comprehensive understanding is desirable in many respects, particularly in view of the possibility to identify new potential therapeutic strategies (DiFrancesco and Borer, 2007; Dunlop et al., 2009; Wickenden et al., 2009; Postea and Biel, 2011; Reid et al., 2012; Shah et al., 2013).

In this review we address the current knowledge of HCN channels properties and their role of dysfunctional behavior in human neurological diseases.

## Epilepsy

Epilepsy is a neurological disorder characterized by recurrent paroxysmal episodes of brain electrical dysfunction. Seizures are caused by an excessive and sudden electrical discharge of neurons of the CNS. Epilepsy is a common disorder, affecting about 4% of the general population, often involving children, and young adults, with a very high cost to society in terms of direct expenses for the public health service and disabilities. Epilepsy affects at least 50 million people worldwide, with a global mortality rate higher than cancer.

The cause of the disease remains unknown in about 60% of cases, which are referred to as “idiopathic.” About 30% of idiopathic epilepsies are inheritable and affect several members of the family. Current research reveals that several types of epilepsy, both familiar, and sporadic, have a genetic component mainly linked to mutations in genes encoding either voltage-gated (Na^+^, Ca^2+^, and K^+^) or ligand-gated (GABAA and cholinergic nicotinic receptor) channels (Avanzini et al., 2007; Mantegazza et al., 2010; Thomas and Berkovic, 2014).

The recognition that certain epileptic syndromes are channelopathies has opened new perspectives in the understanding of the molecular pathophysiology of seizure disorders. Following findings of causative links between epileptic syndromes and mutations in voltage-gated or ligand-gated channels, the family of ion channels involved in epileptogenic channelopathies has grown rapidly. Together with the ion channel types whose involvement has been established early, another family that according to more recent data play a role in the pathogenesis of inheritable epilepsy is the family of HCN channels. Because of their established role in modulating neuronal excitability, a modification of the function of HCN channels is clearly potentially able to cause uncontrolled action potential firing and provide a background setting for the development of epilepsy (Baruscotti et al., 2010).

In the last few years, evidence for a potential relevant role of HCN channels in the pathogenesis of epilepsy has grown considerably. Many results derive from animal models, but a substantial bulk of recent data shows that *HCN* dysfunctional genetic mutations associated with epilepsy are also expressed in human patients.

### Animal Models

Several data supporting a link between functional alteration of HCN channels and epileptogenesis have been obtained by studies of *HCN1* and *HCN2* knock-out mouse models.

For example, cortical excitability and epileptogenesis are enhanced by loss of HCN1 expression and abolishment of I_h_ in *HCN1*-null mice, since lack of this channel increases the dendritic input resistance in cortical neurons, leading to greater synaptic integration, and firing (Huang et al., 2009). In the same *HCN1*-null mouse model, greater dendritic excitability, and temporal summation were also observed in hippocampal CA1 pyramidal neurons (Nolan et al., 2004; Tsay et al., 2007). In another study, in agreement with a role for I_h_ downregulation in epileptogenesis, *HCN2*-deficient mice generated by a global knock-out model were shown to exhibit spontaneous absence seizures (Ludwig et al., 2003). Generalized spike–wave absence seizures were also observed in spontaneous *HCN2* mutant (*apathetic*) mice, where channel proteins are truncated at the C terminus and poorly expressed (Chung et al., 2009). Moreover, different genetic models of absence epilepsy such as WAG/Rij and GABAAγ2(R43Q) revealed an altered expression of HCN1 associated with the occurrence of seizures (Kole et al., 2007; Phillips et al., 2014).

The remodeling of HCN channels expression also provides an important contribution to the epileptogenic network and seizure production. In the immature animal brain, sustained febrile seizures (FS) increase neuronal excitability and the predisposition to develop epilepsy in adults, by modifying the membrane expression of HCN isoforms 1 and 2 (Dube et al., 2000; Brewster et al., 2002). Seizures provoke a prolonged remodeling of HCN1 and HCN2 expression, supporting the presence of a transcriptional channelopathy involving HCN channels which contributes to the development of epilepsy (Richichi et al., 2008). Similar observations can be made in the epileptic human brain tissue, where the expression of HCN channels is altered, indicating that the HCN isoforms are dynamically regulated in humans *in vivo*, as well as in the experimental hippocampal epilepsy (Bender et al., 2003).

In accordance with the observations above, different models of temporal lobe epilepsy (TLE), the most frequent form of partial epilepsy in humans, revealed a significant downregulation of HCN channels expression following pharmacological status epilepticus (SE), with increased neuronal excitability. This could have important implications for both the process of epileptogenesis and maintenance of the epileptic state (Jung et al., 2007, 2011; Powell et al., 2008).

### Human Patients

Despite the availability of a substantial amount of experimental data on animal models supporting the role played by HCN channels in the pathogenesis of epilepsy, evidence from human patients is still limited.

Tang et al. (2008), published a pilot study of 84 patients with a non-inheritable form of idiopathic generalized epilepsy (IGE) and searched for mutations in *HCN1* and *HCN2* in this cohort. They identified, among others, a single point mutation in the C-linker region of HCN2, affecting a highly conserved and functionally relevant residue (R527Q). However, a conclusive proof that this mutation is causative was lacking, since functional studies did not show statistically significant differences between the properties of mutant and wild-type channels. Dibbens and colleagues later reported that in a population of children with FS and genetic epilepsy with FS plus (GEFS+), the rate of appearance of an *HCN2* variant characterized by a triple proline deletion (delPPP, p.719–721) was significantly higher than in the general population. Heterologous expression studies indicated that delPPP channels carry a 35% larger I_h_ current than wild-type channels, suggesting that delPPP behaves as a gain-of-function mutation (Dibbens et al., 2010). However, while statistical significance is a necessary condition to demonstrate a link between mutation and diseased phenotype, the study did not address how the mutation alters the functional properties of channels and how they impact neuronal excitability.

**FIGURE 1:**
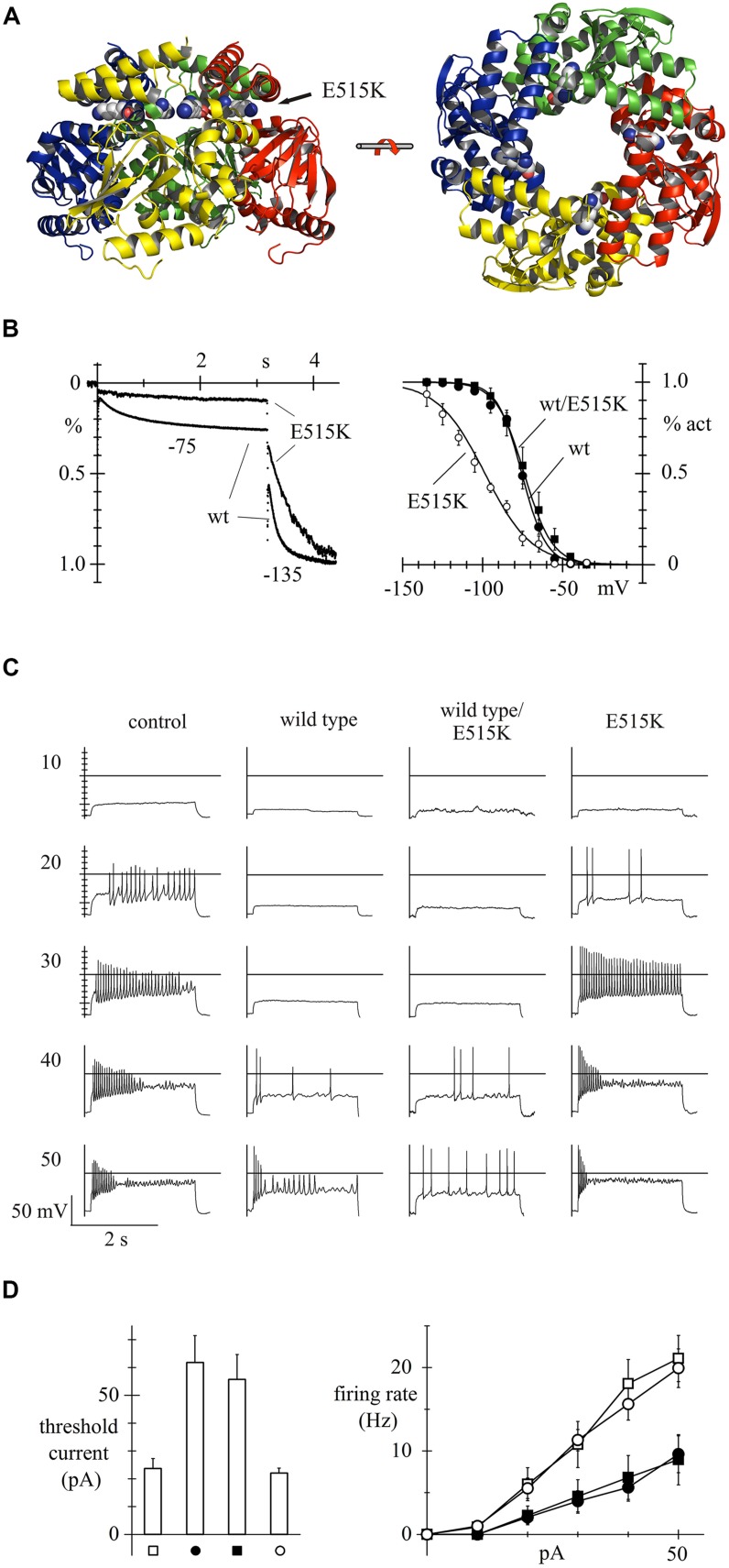
**Human HCN2 E515K mutation and its effect on neuronal excitability.** 3D structure of hHCN2 C-linker and cyclic nucleotide binding domain (CNBD) tetrameric domains based on X-ray crystallographic data (Zagotta et al., 2003) and plotted as ribbon representation. Views shown are perpendicular (left) and parallel (right) to fourfold axis; E515 residues are drawn as space filling plots; the E515K mutation is located in the C-linker region **(A)**. When overexpressed in neonatal rat cortical neurons, the E515K homozygous mutation leads to a large decrease of I_h_ in the voltage range of current activation (left), due to a large negative shift of the activation curve (right), when compared to both the wild-type (wt) and the heterozygous mutation channel (wt/E515K) **(B)**. Measurement of the firing rate due to injection of various degrees of depolarizing currents (range 10–50 pA) shows that neonatal rat cortical neurons transfected with either wild type or heterozygous wild-type/mutant channels respond with a much-reduced excitability than neurons transfected with empty vectors (controls) or with homozygous E515K mutant channels **(C)**. Mean threshold current required to trigger action potential firing and mean rate of firing recorded upon current injection in control, wt, wt/E515K, and E515K cells. Neuronal models transfected with homozygous E515K mutant channels, like empty vectors, show a significantly lower threshold current to trigger neuronal action potential and a higher rate of firing action potential, upon depolarizing current injection **(D)**. Data from (DiFrancesco et al., 2011), with permission.

In a more recent study based on *HCN* candidate-gene screening of a small cohort of patients (*n* = 113), our group characterized for the first time a loss-of-function *HCN2* mutation that could be shown from functional studies to be directly involved in human epilepsy (**Figure 1**). This study led to the identification, in a generalized epilepsy patient, of a homozygous recessive mutation located in the C-linker region of HCN2 (E515K; **Figure 1A**) that determines an almost complete loss of activity of the mutated channel, leading to a significant increase in the activity of neuronal discharge, and of neuronal excitability (DiFrancesco et al., 2011). As shown in **Figure 1B**, homozygous expression of E515K mutated channels in acutely isolated newborn rat cortical neurons led to a large negative shift of the I_h_ current activation curve relative to wild-type channels, while heterozygous expression of wild type/E515K channels did not (**Figure 1B**), indicating that only recessive inheritance of the mutation will display loss-of-function properties. In the neuronal model, following administration of increasing doses of depolarizing current (range 10–50 pA), the transfection of both wild type and heterozygous mutant channel reduced frequency discharge, compared to control conditions (empty-vector transfection) and transfection of homozygous mutant channels (**Figure 1C**). These functional data confirm that HCN2 expression stabilizes excitability, and that removal of HCN2 contribution in homozygous E515K variants is associated with increased excitability, a condition predisposing to epilepsy.

Later work from Nakamura and colleagues reported the finding in two unrelated patients with FS of a mutation in *HCN2* (S126L) which endowed channels with a higher than normal temperature sensitivity. Functional investigation showed that the mutation leads to an increased I_h_ availability at high temperatures (38°C), which may contribute to hyperthermia-induced neuronal hyperexcitability in FS (Nakamura et al., 2013).

More recently, using an exome sequencing approach, *de novo* mutations in *HCN1* have been identified for the first time in early infantile epileptic encephalopathy (EIEE; Nava et al., 2014a). EIEE is a severe disease affecting children within the first year of life which resembles the spectrum of Dravet syndrome and had been previously associated with mutations in *SCN1A* sodium channel and *PCDH19* (Depienne et al., 2009a,b). According to the authors, the mutated HCN1 residues associated with EIEE are all localized in the intracellular part of the channel, although one of them (S272) lies apparently well within the S4 domain according to previously reported data (Santoro et al., 1998). Interestingly, *HCN1* exon deletions have been previously reported in patients with autism spectrum disorders, but without epilepsy (Nava et al., 2014b). The electrophysiological characterization of the mutations revealed for some of them a gain-of-function effect that could play a dominant-negative effect, interfering with the function of the remaining *HCN1* alleles. Taken together, these data suggest that point mutations altering HCN1 channel function are poorly tolerated and predispose to neuronal hyperexcitability, but are insufficient by themselves to cause seizure development. This is supported by data from experimental animal models of global HCN1 knockout, characterized by a predisposition to the development of epilepsy, but without spontaneous seizures (Huang et al., 2009; Santoro et al., 2010). These observations suggest that *de novo HCN1* mutations are associated with EIEE, a particularly severe neurological phenotype, while there is still no evidence for the involvement of *HCN1* in IGE (Tang et al., 2008; Dibbens et al., 2010; DiFrancesco et al., 2011).

**FIGURE 2:**
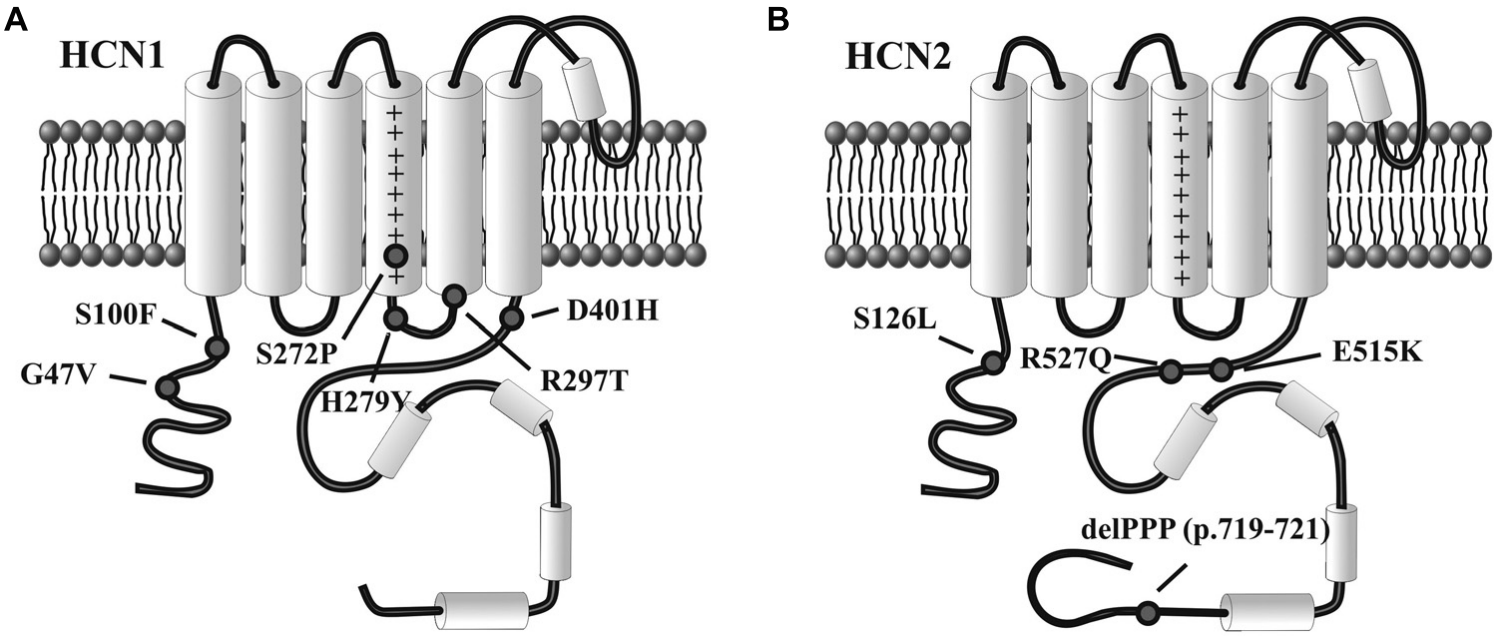
**Variants of HCN1 and HCN2 channels associated with epilepsy.** Diagram representation of single human HCN1 **(A)** and HCN2 channel subunits **(B)** showing the approximate position of mutations linked to infantile epileptic encephalopathy (HCN1) or inheritable forms of epilepsy (HCN2), as reported in the literature. See text for further explanation.

Even if exhaustive genetic studies on a large cohort of patients have not yet been performed, according to these preliminary results (**Table 1**), *HCN1* mutations seem to involve infantile, severe, and progressive epileptic encephalopathies, while *HCN2* appear to affect patients with normal intellective level, without evidence for a progressive disease. Interestingly, there is no evidence yet for the involvement in epilepsy of dysfunctional mutations in *HCN4*, despite this isoform is widely expressed in the human brain. **Figure 2** illustrates the rough sequence positions of mutations of HCN1 and HCN2 channels so far proposed to be associated with epilepsy.

**Table 1:** Mutations of *HCN1* and *HCN2* in human epilepsy.

Gene	Mutations	Phenotype	Biological effect	Reference
*HCN1*	G47V S100F S272P H279Y R297T D401H	Early infantile epileptic encephalopathy (Dravet-like syndrome)	Gain-of-function mutations with possible dominant-negative effect (S100F, S272P, R297T)	Nava et al. (2014a)
*HCN2*	R527Q	Idiopathic generalized epilepsy	No significant variation of I_h_ current	Tang et al. (2008)
	delPPP	Febrile seizures (FS); genetic epilepsy with FS plus (GEFS+)	Gain-of-function mutation	Dibbens et al. (2010)
	E515K	Idiopathic generalized epilepsy	Loss-of-function mutation	DiFrancesco et al. (2011)
	S126L	FS	Temperature-dependent shift of HCN2 channel kinetics	Nakamura et al. (2013)

### Hcn-Accessory Proteins

Dysfunctional behavior of the I_h_ current can also be mediated by changes in the HCN-accessory proteins. This is a set of proteins (such as MiRP1, Filamin A, TRIP8b, Caveolin 3, tamalin, Mint2, S-SCAM, KCR1) that affect the correct function of HCN ion channels by interaction at different levels (Yu et al., 2001; Gravante et al., 2004; Kimura et al., 2004; Santoro et al., 2004, 2009; Barbuti et al., 2007b; Michels et al., 2008; Biel et al., 2009; Noam et al., 2014). It is known for example that the targeted deletion of MiRP1 leads to the downregulation of HCN channel function, which in turn is associated with increased excitability in neurons (Ying et al., 2012). A more direct confirmation of functional interaction between channels and ancillary proteins comes from a study investigating a single patient with long QT syndrome (LQT6) who carried the M54T mutation in MiRP1 (Nawathe et al., 2013). Since this patient was also symptomatic for bradycardia, a condition not normally found in LQT patients, the study explored possible changes in HCN function and found that the M54T mutation indeed causes the HCN4 current density to decrease by about 80%, in agreement with the patient’s bradycardia.

To our knowledge there is no established evidence so far for a direct link between defective HCN accessory proteins and neuronal hyperexcitability in epilepsy patients, although the possibility for such a dependence is likely to be significant.

### Anti Epileptic Drugs

An observation emphasizing the clinical relevance of the concept of HCN-dependent epilepsy is that some anti-epileptic drugs (AEDs) currently used for the treatment of patients have been reported to modify the activity of the I_h_ current. For example lamotrigine, widely used in clinical practice, reduces action potential firing and excitability in dendrites as a result of an increased I_h_, which provides an important potential mechanism for its antiepileptic action (Poolos et al., 2002). In addition, the antiepileptic drug gabapentin increases I_h_ in CA1 pyramidal neurons (Surges et al., 2003) and acetazolamide, an inhibitor of carbonic anhydrase that has been used to treat epilepsy, can increase I_h_ through intracellular alkalinization (Munsch and Pape, 1999). These data confirm the role played by the I_h_ current in the epileptogenic process.

## Pain

The sensation of pain is evoked when the body is exposed to a potentially dangerous stimulus. This determines nerve membrane depolarization and generation of action potentials from peripheral nociceptors to CNS. Pain can generally be divided into inflammatory and neuropathic.

Acute inflammatory pain has the crucial role of protecting the body from external damage, thus lowering the risk of further damage or infection of an injured area. It is triggered by the release of inflammatory factors, such as prostaglandin E_2_ (PGE_2_) and enhances the ability of nociceptive nerve terminals to generate action potentials, thus causing local hypersensitivity (Linley et al., 2010). Typically, when the stimulus ceases, nociceptive pain perception disappears. However, in certain circumstances, such as diabetes, or herpes zoster infection, inflammatory pain becomes chronic, and seriously impairs the quality of life of patients.

Neuropathic pain is a long-lasting pain state caused by nerve injury, poorly treated by pharmacological drugs. Patients frequently complain of painful spontaneous sensations, evoked by normally harmless stimuli. While a major role of CNS sensory processing is likely in neuropathic pain transmission, the condition of the injured peripheral nerve is critical for its initiation and maintenance.

There is increasing evidence demonstrating a crucial role played by the class of HCN ion channels in starting and controlling firing frequency of action potentials responsible for pain (Jiang et al., 2008). Although the HCN1 isoform is one of the pharmacological targets of the potent and widely used anesthetic propofol (Tibbs et al., 2013), its contribution to the transmission of pain seems to be limited. Recent experimental data have indeed demonstrated the strategic role played by HCN2 in both inflammatory and neuropathic pain, suggesting the selective blocking of its activity as a potential target for the treatment of pain.

### Inflammatory Pain

It is established that the inhibition of the I_h_ current, by drugs blocking HCN channels activity in a non-selective manner, prevents inflammatory pain generated in various animal models by different stimuli (Luo et al., 2007; Dunlop et al., 2009; Takasu et al., 2010). However, these results do not allow to explain the contribution of the specific channel isoforms involved in pain. Selective genetic deletion of different HCN isoforms recently clarified this important aspect.

According to studies in an *HCN1* KO model, this channel isoform does not appear to play a crucial role in the transmission of inflammatory pain (Momin et al., 2008). *HCN2* global KO models have not been tested for the studies of pain, since they are associated with severe neurological phenotypes, such as generalized epilepsy and ataxia, and are not prone to behavioral studies (Ludwig et al., 2003; Chung et al., 2009).

Recently, a genetic approach using the selective deletion of HCN2 in the Na_V_1.8 sensory neurons has contributed to clarify the important role played by HCN2 in the generation and maintenance of pain. The study showed that the perception of pain secondary to the administration of different inflammatory stimuli was lost in *HCN2* KO mice, while the reaction to acute pain in the absence of inflammation remained unaltered. These data strongly suggest that block of HCN2 can be considered as a putatively very useful strategy for the treatment of inflammatory pain, without the adverse effect of reducing the perception of the normal acute stimulus (Emery et al., 2011, 2012).

### Neuropathic Pain

Non-selective pharmacological block of HCN channels also inhibits neuropathic pain, suggesting their involvement in pain perception caused by direct nerve injury. The genetic deletion of HCN1 partially reduces the neuropathic pain determined by direct damage on the nerve (Momin et al., 2008), while the contribution of HCN2 appears to be more substantial. The animal model with a selective KO of *HCN2* in Na_V_1.8-expressing peripheral neurons shows the absence of hyperalgesia following the application of different nociceptive stimuli that determine nerve injury. These results support the notion that the HCN2 isoform is a key factor in initiating neuronal excitability following nerve damage and in the maintenance of neuropathic pain. Accordingly, the selective inhibition of HCN2 activity could be expected to represent an interesting specific target for the treatment of this debilitating condition, widespread in the general population. However, given the key role played by HCN2 in the human CNS (DiFrancesco et al., 2011), any specific HCN2-blocking drug used as a therapy against neuropathic pain should be able to perfuse the peripheral nervous system, without passing across the blood brain barrier, in order to avoid serious neurological adverse consequence, such as seizures.

It is also worth noticing that ivabradine, the unique clinically approved non-selective HCN blocker used for coronary heart disease and heart failure (DiFrancesco and Camm, 2004; DiFrancesco and Borer, 2007) has been reported to reduce mechanical allodynia, inflammatory and neuropathic pain in animal models, further supporting the involvement of HCN channels in pain transmission (Noh et al., 2014; Young et al., 2014). However, until more isoform-specific drugs are commercially available, clinical use of ivabradine in the treatment of pain is obviously hindered by the bradycardic effect due to block of HCN4 activity in the heart.

## Parkinson’s Disease

Parkinson’s disease (PD) is one of the most common neurodegenerative disease, characterized by progressive motor symptoms such as bradykinesia, rigidity, and resting tremor (Hammond et al., 2007; Ali and Morris, 2015). Progressive degeneration of dopamine (DA) neurons in the substantia nigra pars compacta (SNc) is the main etiology of PD.

HCN2 and HCN4 channels are highly expressed in midbrain DA neurons, where they participate in the regulation of network spontaneous activity (Mercuri et al., 1995; Santoro et al., 2000; Notomi and Shigemoto, 2004; Chu and Zhen, 2010).

Recent data acquired from genetic and pharmacological animal models of PD reported a progressive downregulation of HCN channel activity following DA depletion and DA neuronal loss, although channel protein expression did not appear to be affected (Good et al., 2011). Interestingly, the delivery of HCN subunits restored pacemaking activity and reduced burst spiking, even if the motor disability induced by DA depletion was not reversed (Chan et al., 2011). A role of HCN channel downregulation in PD is also supported by a computational model of the activity of the globus pallidus, which suggests a possible mechanism for the emergence of parkinsonian activity (Merrison-Hort and Borisyuk, 2013).

Recently, Masi et al. (2013) reported novel evidence for a possible influence of HCN channel dysfunction in the pathogenesis of human PD. They showed that MPP^+^, a potent parkinsonizing agent well known as a blocker of the mitochondrial complex I, also leads to a dose-dependent inhibition of I_h_ in SNc DA neurons. The electrophysiological analysis of this pharmacological model revealed a reduced spontaneous activity of I_h_ in SNc DA neurons, characterized by an increased responsiveness toward synaptic excitation, possibly contributing to the pathogenesis of human PD.

## Conclusion

HCN channels play a fundamental role in the control of neuronal excitability and network activity in the nervous system. Accumulating evidence supports the view that modifications in the HCN physiological function contribute to the pathogenic mechanism leading to certain neurological diseases in humans such as epilepsy, pain, and, as recently emerged, PD.

Although it is now clear that HCN channels play an important role in the pathogenesis of epilepsy, the precise mechanisms underlying their role in this common disease still remains to be elucidated. Thorough genetic studies of large populations of epileptic patients, able to associate the various forms of epilepsy (i.e., absence, IGE, TLE, various type of epileptic encephalopathies) to specific alterations of the channels, have yet to be performed. However, preliminary results today available suggest that *HCN1* point mutations leading to channel dysfunction are associated with severe and progressive epileptic diseases, while *HCN2* mutations account for milder generalized phenotypes, without evidence of a progressive feature. Interestingly, there are no mutations of HCN channels reported in partial epilepsies TLE, although the experimental data from animal models allow to hypothesize their presence in humans. It will certainly be necessary to better understand the role played by dysfunctional HCN channels in predisposing to the development of the epileptogenic action potential. Understanding these aspects will contribute to identify novel potential therapeutic strategies for the treatment of epilepsy.

It is also important to note that the involvement of HCN channels in a wide variety of neuronal processes including sensory signal transduction, dendritic integration, synaptic plasticity, pacemaking, and network oscillations, motor learning, and others (Robinson and Siegelbaum, 2003) makes them likely contributors, when defective, to pathological behavior. As a natural consequence of their contribution as neurological disease-causing factors, HCN channels have long been considered an attractive therapeutic target (Postea and Biel, 2011; Reid et al., 2012; Shah et al., 2013). It is interesting to note in this context that, while most epilepsy-linked HCN mutations shown to generate hyperexcitability are loss-of-function (Lewis and Chetkovich, 2011), reduction of I_h_ activity is known to reduce chronic pain (Emery et al., 2012; Young et al., 2014). The reason for this apparently paradoxical effect is that neuronal excitability depends both on input membrane resistance and resting membrane voltage, whose I_h_-linked modifications affect excitability in opposite ways (Poolos et al., 2002). It will be crucial in the near future to clarify the details of HCN-associated excitatory vs. inhibitory effects, in order to identify specific pharmacological strategies for these two clinical conditions, both extremely common in the general population and poorly treated by conventional drug therapy, and often characterized by multiple side effects.

The understanding of the processes rendering HCN channels dysfunctional in human neurological diseases will provide in the future further important insight into the molecular mechanisms responsible for the disease, a concept likely to be applicable to a larger number of ion channelopathies than is known to date. While studies of animal models can clearly provide essential basic information, it is highly desirable that further investigation is focused on the identification of the role played by HCN channelopathies in human diseases. These studies should be eventually aimed at the development of selective pharmacological strategies specifically targeting HCN channels for the treatment of the neurological diseases.

## Conflict of Interest Statement

The authors declare that the research was conducted in the absence of any commercial or financial relationships that could be construed as a potential conflict of interest.
